# Sensitization to Airborne Fungal Allergens Associates with Asthma and Allergic Rhinitis Presentation and Severity in the Singaporean/Malaysian Population

**DOI:** 10.1007/s11046-021-00532-6

**Published:** 2021-07-13

**Authors:** Yang Yie Sio, Sze Lei Pang, Yee-How Say, Keng Foo Teh, Yi Ru Wong, Smyrna Moti Rawanan Shah, Kavita Reginald, Fook Tim Chew

**Affiliations:** 1 Present Address: Department of Biological Sciences, National University of Singapore, Singapore, Singapore; 2 Department of Biomedical Science, Faculty of Science, Universiti Tunku Abdul Rahman (UTAR) Kampar Campus, Kampar, Perak, Malaysia; 3 Department of Biological Sciences, School of Science and Technology, Sunway University, Subang Jaya, Malaysia

**Keywords:** Allergy, Fungi, Sensitization, *Aspergillus*, *Curvularia*

## Abstract

Fungal spores and conidia are the major components of total airspora in the tropical Asia environment, and their sensitization patterns are often associated with allergic diseases such as asthma, allergic rhinitis (AR), and atopic dermatitis. Hence, we recruited a cross-sectional cohort of 9223 Singapore/Malaysia Chinese adults and assessed their sensitization against *Curvularia lunata* allergen using the skin prick test approach. A subset of this cohort (*n* = 254) was also screened for specific Immunoglobulin E (sIgE) titers against a panel of 11 fungal allergens. We found significant association of *Curvularia lunata* sensitization with the risk of asthma (OR = 1.66, 95% CI: 1.17–2.33*; p* = 0.00391) and AR (OR = 1.69, 95% CI: 1.18–2.41; *p* = 0.00396). Among asthmatic patients (*n* = 1680), *Curvularia lunata* sensitization also increased frequencies of wheezing symptoms (OR = 1.81, 95% CI: 1.05–2.96; *p* = 0.0239), general practitioner/specialist visits (OR = 2.37, 95% CI: 1.13–4.61; *p* = 0.0157), and other asthma-related exacerbation events (OR = 2.14, 95% CI: 1.04–4.10; *p* = 0.0289). In our serum cohort, sensitization to *Aspergillus* spp. was the most common fungal sensitization, with 23.6% (*n* = 60) had a class 3 and above sensitization (positive sensitization; sIgE titers of > 3.5 kU/L) against this allergen. Increasing sIgE titer against *Aspergillus* spp. was also correlated with increased AR risk and AR-related symptoms. In conclusion, our findings emphasize an important role of fungal sensitization in the manifestations of asthma and AR in the Southeast Asian Chinese population.

**Supplementary Information:**

The online version contains supplementary material available at (10.1007/s11046-021-00532-6).

## Introduction

The worldwide prevalence of allergic diseases, including asthma, allergic rhinitis (AR), and atopic dermatitis (AD), has increased over the past decades [1–3]. Sensitizations to dust mites, pollen, and spores have been previously indicated as major triggers of these allergic diseases [4]. AR has been considered as a post-industrial revolution epidemic due to increase in urbanization air pollution [5]. The ongoing fourth industrial revolution will continue to exacerbate allergic diseases due to air pollution and climate change [6]. In the tropical urban environment of Singapore, multiple fungal species including *Cladosporium* spp., *Didymosphaeria* spp., *Curvularia* spp., *Drechslera* spp., and *Pithomyces* spp. were the most dominant fungal spore type in the total outdoor airspora and their spores densities were affected by temperature and relative humidity [7].

Here, we report the association of airborne fungal sensitization with allergic disease susceptibility and severity in a Southeast Asian Chinese population. Using a cross-sectional cohort of 9923 Singapore/Malaysia Chinese adults, we assessed their *Curvularia lunata* sensitization profile using skin prick test (SPT) and compared across their asthma-, AR-, or AD-related phenotypes. Using a subset of serum samples, we also evaluated specific Immunoglobulin E (specific IgE or sIgE) titers against multiple fungal allergens presented in the tropical Asia environment and associated them with AR-related phenotypes.

## Materials and Methods

### Study Design

The current study belonged to a part of our ongoing epidemiology study on allergic diseases. Participants were recruited from three locations, including the National University of Singapore (NUS), Universiti Tunku Abdul Rahman (UTAR), and Sunway University (SU), Malaysia. Recruitments were conducted from Aug 2005 to Sep 2019 in NUS, from Feb 2016 to Oct 2018 in UTAR, and on Nov 2019 in SU. Participants were requested to complete an investigator-administered questionnaire collecting information on demographics and medical history, which was based on the Allergic Rhinitis Impact on Asthma (ARIA) [1] and International Study of Asthma and Allergies in Childhood (ISAAC) [8] guidelines.

Participants also underwent a skin prick test (SPT). Our full SPT panel included 2 house dust mite species (*Dermatophagoides pteronyssinus* and *Blomia tropicalis*), *Curvularia lunata*, and Oil palm pollen (*Elaeis guineensis*). These allergens were previously shown to be the most common inhalant allergen presented in the Singapore environment with high allergenicity [7, 9]. Sensitization to house dust mite has already been discussed in our previous publication [10]. We also observed a separate and independent association between sensitization to oil palm pollen and allergic diseases presentation. This will be discussed in a separate publication. In this manuscript, we focused on Curvularia spp. sensitization because this was one of the most abundant outdoor fungal species as mentioned in our previous publication [7].

Ten milliliters of whole blood samples were collected from a subset of participants in our cross-sectional cohort. Serum was then separated via centrifugation at 2600 g and 4 °C for 15 min and measured for sIgE titer against 11 fungal allergens (*Aspergillus* spp., *Candida albicans, Penicillium* spp., *Trichophyton* spp., *Saccharomyces cerevisiae, Fusarium* spp., *Trichoderma viride, Stemphylium botryosum, Cladosporium* spp., *Malazessia furfur,* and *Curvularia* spp.) using the immuno-dot blot assay, as described previously [11–14]. Sensitization of class 3 and above (sIgE titers of > 3.5 kU/L) was considered as positive sensitization. All immune-dot blot measurements were conducted at the same time to avoid batch variation.

Asthma was defined as ever having asthma positively diagnosed by a physician. AR was defined as having at least two major AR-related symptoms that include nasal congestion, rhinorrhea, nasal itching, and sneezing (based on 2008 guidelines set by the ARIA consortium) [1]. Based on the severity of these four AR-related symptoms and an approach that was adopted from the Symptomatic Global Score for seasonal allergic rhinitis [15], a numeric score was also calculated for the AR cases and was categorized into three groups—low: 0–2, mild: 3–4, moderate-severe: > 4. AD was defined as having a persistent itchy rash that affected flexural areas. These allergic conditions (asthma, AR, and AD) were further confirmed by participants having an atopy condition, defined as having a positive SPT reaction toward one of the two common house dust mites, *Dermatophagoides pteronyssinus* or *Blomia tropicalis*. Ethnicity was self-identified through a questionnaire and further confirmed through a previously performed principal component analysis [16].

Association analysis between fungal sensitization and allergic disease-related phenotypes was performed using independent sample *t*-test or logistic regression analysis in the R program version 3.6.1 (R Foundation for Statistical Computing, Vienna, Austria), and *p* < 0.05 was considered as statistically significant.

## Results

### Sensitization to the *Curvularia* is Associated with the Susceptibility and Severity of Asthma and AR

Our cross-sectional cohort comprised 9923 Singapore/Malaysia Chinese adults (age: 21.90 ± 4.93), with asthma, AR, and AD prevalence of 16.9%, 32.3%, and 14.4%, respectively (Supplementary Table 1). In this cohort, the sensitization rate to *Curvularia lunata* was 1.9% (Supplementary Table 1). A positive SPT reaction to the *Curvularia lunata* allergen was significantly associated with an increased susceptibility to asthma and AR, but not AD (Table 1). Among asthmatic cases, *Curvularia lunata* sensitization was also significantly associated with the presence of some recent (past 12 months) asthma symptoms or exacerbation events, which included wheezing, general practitioner/specialist visits, and any asthma-related exacerbation event (Table 1). Besides, when AR patients were classified into three groups based on their symptom scores, a moderate-severe phenotype was also associated with *Curvularia lunata* sensitization, albeit marginally significant (*p* = 0.0555; Table 1). Additionally, individuals with *Curvularia lunata* sensitization also had increased risk of having both asthma and AR conditions, as well as both AR and AD conditions (Table 1).

**Table 1:** Associations of *Curvularia lunata* sensitization with allergic diseases risk and severity in the Singapore/Malaysia Chinese population

	*Curvularia lunata* Positive SPT	*Curvularia lunata* Negative SPT	*p* value	OR (95% CI)
	Case/control (%Case)	Case/control (%Case)		
Asthma (AS)	50/118 (29.8)	1630/6860 (19.2)	0.00391*	1.66 (1.17–2.33)
Wheezing (past 12 months)	13/32 (28.9)	303/1269 (19.3)	0.0239*	1.81 (1.05–2.96)
Daytime Asthma attack (past 12 months)	2/40 (4.8)	38/1194 (3.1)	0.474	1.70 (0.27–5.81)
Nighttime Asthma attack (past 12 months)	2/40 (4.8)	41/1186 (3.3)	0.170	2.35 (0.55–6.86)
School absence due to asthma (past 12 months)	5/37 (11.9)	77/1148 (6.3)	0.240	1.87 (0.59–4.89)
GP/specialist Visits due to Asthma (past 12 months)	11/30 (26.8)	197/1027 (16.1)	0.0157*	2.37 (1.13–4.61)
A and E Admission due to Asthma (past 12 months)	3/39 (7.1)	51/1161 (4.2)	0.537	1.50 (0.34–4.68)
Hospitalization due to asthma (past 12 months)	1/38 (2.6)	19/1145 (1.6)	0.912	1.12 (0.06–5.57)
Any Exacerbation event† (past 12 months)	12/30 (28.6)	213/1012 (17.4)	0.0289*	2.14 (1.04–4.10)
Allergic Rhinitis (AR)	75/54 (58.1)	3126/4043 (43.6)	0.00396*	1.69 (1.18–2.41)
AR symptom scores: low (0–2)	4 (3.1)	155 (2.2)	ref	
AR symptom scores: mild (3–4)	11 (8.5)	452 (6.3)	0.739	1.09 (0.65–1.75)
AR symptom scores: moderate-severe (> 4)	60 (46.5)	2518 (35.1)	0.0555	1.36 (0.99–1.87)
Atopic Dermatitis (AD)	36/125 (22.4)	1392/6792 (17)	0.102	1.37 (0.93–1.98)
AS + AR	24/42 (36.4)	861/3241 (21)	0.0491*	1.70 (0.99–2.85)
AS + AD	11/79 (12.2)	409/4981 (7.6)	0.224	1.52 (0.73–2.86)
AR + AD	22/43 (33.8)	804/3187 (20.1)	0.0174*	1.90 (1.10–3.19)
AS + AR + AD	9/34 (20.9)	261/2597 (9.1)	0.107	1.95 (0.81–4.18)

A and E: accident and emergency department of a hospital; CI: confidence interval; GP: general practitioner; SPT: skin prick test. All data were evaluated based on skin prick test results of the *Curvularia lunata* allergen in a cross-sectional cohort of Singapore/Malaysia Chinese individuals (*n* = 9223). *p* value, odds ratio, and 95% CI were calculated using a logistic regression analysis with adjustment for age and gender

^†^Any exacerbation event includes school absence, GP/specialist visits, A and E admission, or hospitalization due to asthma exacerbation

*p < 0.05 is considered as significant

### Serum sIgE Titers Against *Aspergillus* spp. Correspond with the Outcomes and Severity of AR

**Fig. 1:**
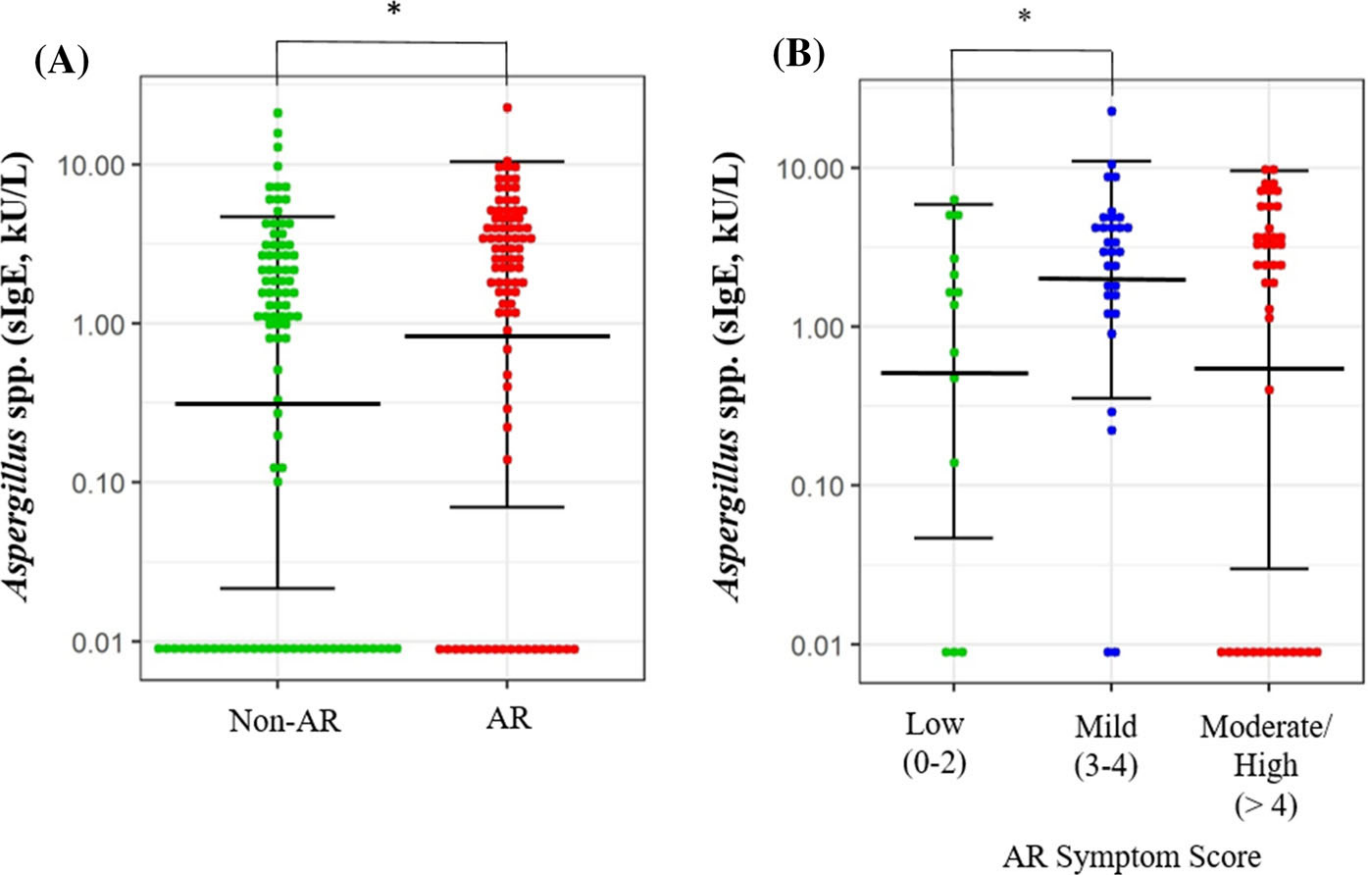
Association of *Aspergillus* spp. sensitization with AR risk and severity in the Singapore/Malaysia Chinese population. Serum sIgE titers specific to *Aspergillus* spp. were compared across **a** AR and **b** AR severity based on test subject’s AR symptom score (low: 0–2, mild: 3–4, and moderate-severe: > 4), using a cross-sectional cohort of Singapore/Malaysia Chinese individuals (*n* = 254). *t-test *p* value < 0.05

Next, we collected 254 individuals’ serum samples from a subset of our cross-sectional cohort (Supplementary Table 1) and measured sIgE titers against 11 common fungal allergens. Positive sensitization to *Aspergillus* spp. was observed to be the most common (23.62%, *n* = 60) among all the tested fungal allergens (Supplementary Fig. 1). Furthermore, significant pairwise correlations were observed across all the sIgE titers against 11 types of fungal allergens tested. (Pearson’s R ranged from 0.283 to 0.721, all *p* < 0.0001). These include the correlation between sIgE titers against *Aspergillus* spp. and *Curvularia* spp. (Pearson’s R = 0.373, *p* < 0.0001, Supplementary Fig. 2). In this serum cohort, we found that individuals with AR generally had significantly higher *Aspergillus* spp.-specific IgE levels compared to controls (*p* = 0.043, Fig. 1a), and those with mild AR symptom scores also had significantly higher sIgE levels than those with low AR symptom scores (*p* = 0.040, Fig. 1b).

## Discussion

In our Southeast Asian Chinese population, we detected significant associations of fungal sensitization with the risk and severity of asthma and AR. Sensitization to *Curvularia lunata* allergen increases the risk of asthma and AR, while also increasing the frequencies of wheezing symptoms and exacerbation events among asthmatic patients. Furthermore, sensitization to *Aspergillus* spp. was the commonest among all tested fungal allergens in our serum cohort, with 23.6% having positive sensitization. Increasing sIgE titer against *Aspergillus* spp. was also correlated with increased AR risk and AR-related symptoms.

Our study focused on Chinese individuals who resided in the tropical environment of Singapore and Malaysia. A previous aerobiological survey has revealed a rich fungal airspora present in the Singapore environment [7]. *Curvularia lunata* is especially prevalent in tropical climatic conditions like Singapore and Malaysia, albeit its spore counts decreased with increases in relative humidity and precipitation [7], while the highest concentrations of *Aspergillus fumigatus* were observed during monsoon season in a southern tropical Indian region [17]. Individuals residing in this region may therefore likely be sensitized to these fungal allergens and subsequently have increased susceptibility to allergic diseases. In agreement with this, a study has shown an association between *Curvularia* spp. sensitization and atopic diseases in the Singapore population [9]. By using a large cohort of Singapore/Malaysia Chinese individuals that is independent of the previous cohort [9], we were able to confirm this association and showed that *Curvularia lunata* sensitization is associated with the risk of asthma and AR, as well as asthma-related symptoms and exacerbation events. Besides, our study has further demonstrated significant associations of serum *Aspergillus* spp.-specific IgE titer with AR risk and severity.

Due to a small sample size of asthma cases in the serum cohort, thus limiting statistical power, we were unable to determine a significant association of sIgE specific to fungal allergens with asthma risk and severity. Nevertheless, *Aspergillus* spp. sensitization was previously associated with frequent asthma exacerbations and increased corticosteroid requirement in the Singapore population with severe asthma [18]. Increasing serum collection from more asthma cases in future may help to confirm this association. Besides, we have not characterized the role of age in fungal sensitization in our study population. This is because our cohort comprises mostly young participants (21.90 ± 4.93 years old for the cross-sectional cohort, supplementary Table 1) that were recruited from three different universities in Singapore and Malaysia. Although the role of age is unclear, a previous study has observed an increased fungal sensitization rate in subjects of higher age [9]. Further study is therefore required to validate this observation.

In conclusion, the current study has characterized fungal sensitization patterns and showed their association with the development of allergic diseases in the Southeast Asian Chinese population. Further characterizations of these fungal allergenic components may provide a better understanding of the manifestations of allergic diseases in this region, which allow improvement of the current diagnostic and therapeutic approaches of allergic diseases.

## Supplementary Information

Supplementary file 1 (DOCX 96 kb)

Supplementary file 2 (DOCX 208 kb)

Supplementary file 3 (DOCX 19 kb)

## Data Availability

All data generated, analyzed, and included in this study are available from the corresponding author (Chew Fook Tim).
