# Supplementary material for: Sensitization to Airborne Fungal Allergens Associates with Asthma and Allergic Rhinitis Presentation and Severity in the Singaporean/Malaysian Population
Source: Mycopathologia. 2021 Jul 13;186(5):583–8. doi: 10.1007/s11046-021-00532-6 (PMC8536550; doi:10.1007/s11046-021-00532-6)
Supplement: Supplementary file 1 — (DOCX 96 kb) [file 11046_2021_532_MOESM1_ESM.docx]

**
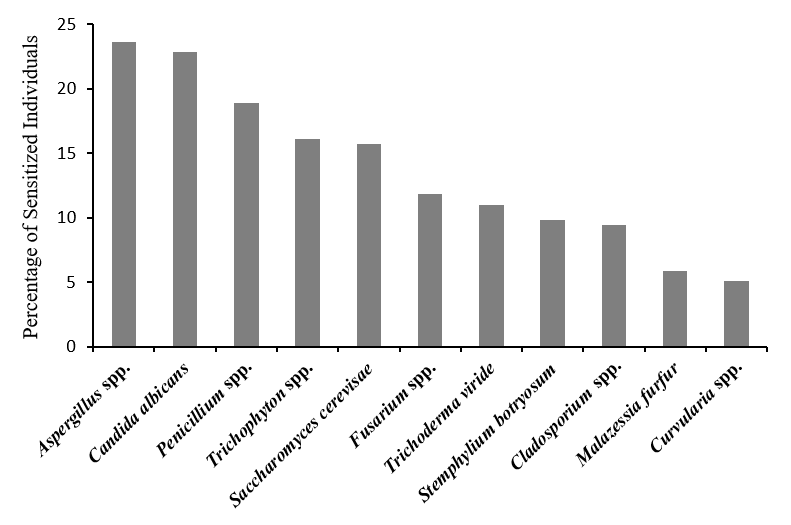
**

**Supplementary Figure S1**: Prevalence of fungal sensitization in the Singapore/Malaysia Chinese Population. Serum sIgE titers against 11 fungal allergens were assessed using a cross-sectional cohort of Singapore/Malaysia Chinese individuals (*n* = 254). A Class 3 and above sensitization to the allergen of interest is considered a positive sensitization (sIgE titers of > 3.5kU/L).
