# Supplementary material for: Sensitization to Airborne Fungal Allergens Associates with Asthma and Allergic Rhinitis Presentation and Severity in the Singaporean/Malaysian Population
Source: Mycopathologia. 2021 Jul 13;186(5):583–8. doi: 10.1007/s11046-021-00532-6 (PMC8536550; doi:10.1007/s11046-021-00532-6)
Supplement: Supplementary file 2 — (DOCX 208 kb) [file 11046_2021_532_MOESM2_ESM.docx]

**
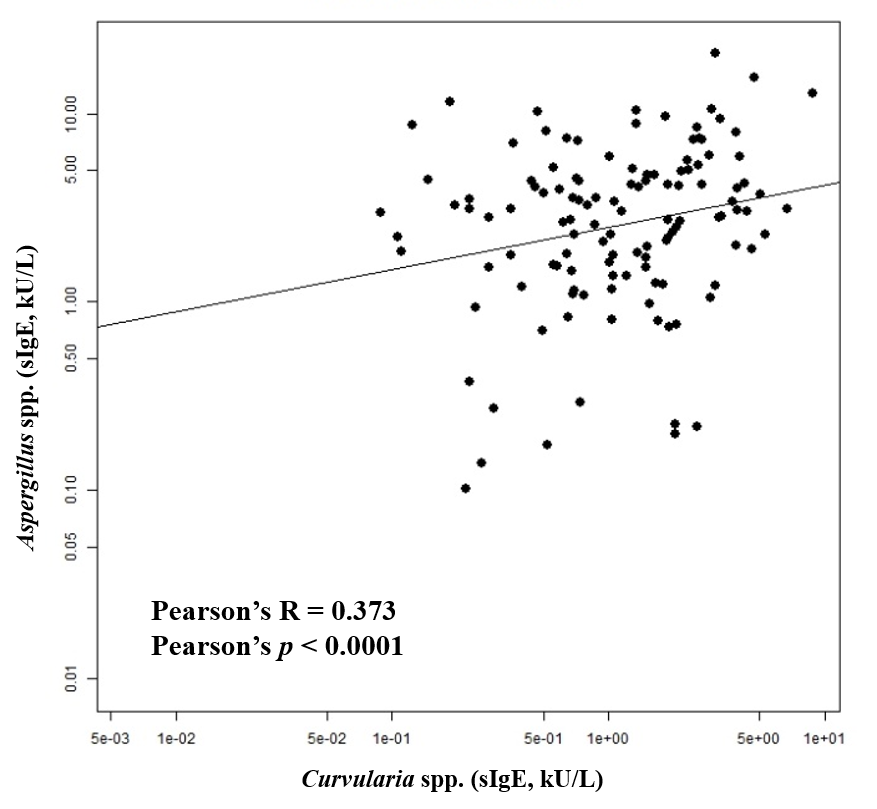
**

**Supplementary Figure S2**: Pairwise correlation plot of serum sIgE titers against *Aspergillus* spp. and *Curvularia* spp. allergens. Pearson’s correlation coefficients were calculated using a cross-sectional cohort of Singapore/Malaysia Chinese individuals (*n* = 254).
