# Supplementary material for: Sensitization to Airborne Fungal Allergens Associates with Asthma and Allergic Rhinitis Presentation and Severity in the Singaporean/Malaysian Population
Source: Mycopathologia. 2021 Jul 13;186(5):583–8. doi: 10.1007/s11046-021-00532-6 (PMC8536550; doi:10.1007/s11046-021-00532-6)
Supplement: Supplementary file 3 — (DOCX 19 kb) [file 11046_2021_532_MOESM3_ESM.docx]

**Supplementary Table S1**: Demographics of the Singapore/Malaysia Chinese cohort.

|  | Cross-sectional Cohort | Serum Cohort* |
| --- | --- | --- |
| Total (n) | 9923 | 254 |
| Gender: Male (*n*, %) | 4161 (41.9) | 147 (57.9) |
| Female (*n*, %) | 5677 (57.2) | 106 (41.7) |
| Age (Year, Mean ± 1SD) | 21.90 ± 4.93 | 20.24 ± 2.47 |
| Asthma (*n*, %) | 1680 (16.9) | 35 (13.8) |
| Allergic Rhinitis/AR (*n*, %) | 3201 (32.3) | 86 (33.9) |
| Atopic Dermatitis/AD (*n*, %) | 1428 (14.4) | 30 (11.8) |
| Sensitization to *Curvularia lunata* (SPT positive) | 192 (1.9) | 6 (2.4) |

SD: standard deviation; SPT: skin prick test. *Serum cohort is a subset of the cross-sectional cohort.
